# Synchrony between sensory and cognitive networks is associated with subclinical variation in autistic traits

**DOI:** 10.3389/fnhum.2015.00146

**Published:** 2015-03-23

**Authors:** Jacob S. Young, David V. Smith, Christopher G. Coutlee, Scott A. Huettel

**Affiliations:** ^1^Pritzker School of Medicine, University of ChicagoChicago, IL, USA; ^2^Department of Psychology, Rutgers UniversityNewark, NJ, USA; ^3^Center for Cognitive Neuroscience, Duke UniversityDurham, NC, USA; ^4^Department of Psychology and Neuroscience, Duke UniversityDurham, NC, USA

**Keywords:** autism quotient, executive control network, dual regression, independent component analysis, functional connectivity, face processing

## Abstract

Individuals with autistic spectrum disorders exhibit distinct personality traits linked to attentional, social, and affective functions, and those traits are expressed with varying levels of severity in the neurotypical and subclinical population. Variation in autistic traits has been linked to reduced functional and structural connectivity (i.e., underconnectivity, or reduced synchrony) with neural networks modulated by attentional, social, and affective functions. Yet, it remains unclear whether reduced synchrony between these neural networks contributes to autistic traits. To investigate this issue, we used functional magnetic resonance imaging to record brain activation while neurotypical participants who varied in their subclinical scores on the Autism-Spectrum Quotient (AQ) viewed alternating blocks of social and nonsocial stimuli (i.e., images of faces and of landscape scenes). We used independent component analysis (ICA) combined with a spatiotemporal regression to quantify synchrony between neural networks. Our results indicated that decreased synchrony between the executive control network (ECN) and a face-scene network (FSN) predicted higher scores on the AQ. This relationship was not explained by individual differences in head motion, preferences for faces, or personality variables related to social cognition. Our findings build on clinical reports by demonstrating that reduced synchrony between distinct neural networks contributes to a range of subclinical autistic traits.

## Introduction

Navigating our social environment requires us to infer the thoughts, intentions, and goals of others (Saxe, 2006). These social computations are disrupted in a host of psychopathologies (Lyon et al., 1999; Couture et al., 2006; Zucker et al., 2007), particularly autism spectrum disorders (ASD; Pelphrey et al., 2004; Oberman and Ramachandran, 2007). Individuals with ASD exhibit a range of altered attentional, social, and affective functions, as evident with varying levels of severity in the subclinical population (Gerdts and Bernier, 2011). Recent work has attempted to quantify variability in autistic traits—from subclinical to clinical levels—using the Autism-Spectrum Quotient (AQ; Baron-Cohen et al., 2001). This questionnaire provides a broad assessment of multiple areas, including social skills, attention switching, attention to detail, communication, and imagination (Baron-Cohen et al., 2001). Although high scores on the AQ can provide useful diagnostic information regarding ASD (Woodbury-Smith et al., 2005), variation in AQ primarily reflects the extent of autistic traits within an individual, thereby providing information regarding individual differences in autistic traits, even within a subclinical sample (Baron-Cohen et al., 2001).

Understanding the neural bases of variability in the AQ and ASD has emerged as a focal point across several studies in social neuroscience (Adolphs, 2010). Studies employing tasks requiring social cognition and attentional control have implicated a number of brain areas—particularly the amygdala (Ashwin et al., 2007), fusiform gyrus (Dziobek et al., 2010), anterior cingulate cortex (Dichter et al., 2009), and the temporo-parietal junction (Lombardo et al., 2011; von dem Hagen et al., 2011)—in the pathophysiology of autism. Building on these observations, other work has highlighted the importance of underconnectivity—evident as changes in white matter tracts or reduced synchrony in functional magnetic resonance imaging (fMRI) data—within these brain systems (Just et al., 2004, 2007); in other words, greater severity in autistic traits, whether clinical or subclinical, can be associated with reduced connectivity within specific brain systems. Recent findings suggest that reduced connectivity within resting-state networks containing the amygdala is associated with greater severity in autistic traits (von dem Hagen et al., 2013). Such results lead to the specific hypothesis that the interactions between different resting-state networks—particularly during the processing of socially-relevant stimuli such as faces, which convey important social information regarding other individuals (Little et al., 2008)—may be disrupted by variation in autistic traits.

**Figure 1:**
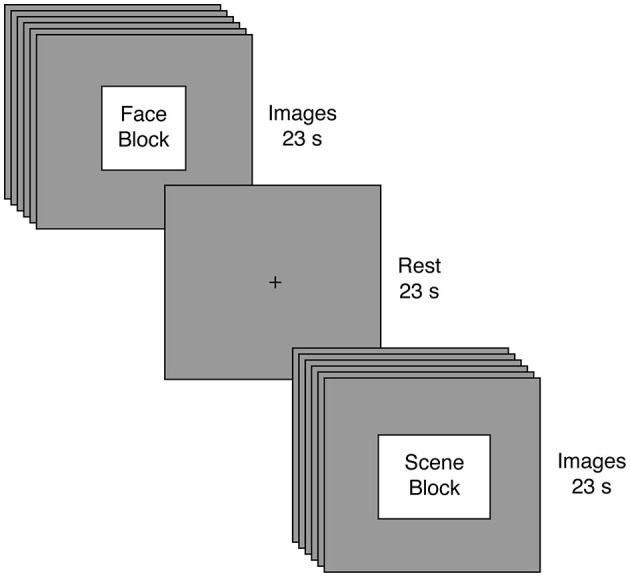
**Experimental Task**. Participants engaged in a passive visual stimulation paradigm involving alternating blocks of faces and scenes. Blocks were 23 s in duration and comprised six different images, with each image presented for 3 s and followed by a 1 s fixation cross. Following every block of visual stimulation, participants rested for 23 s.

**Figure 2:**
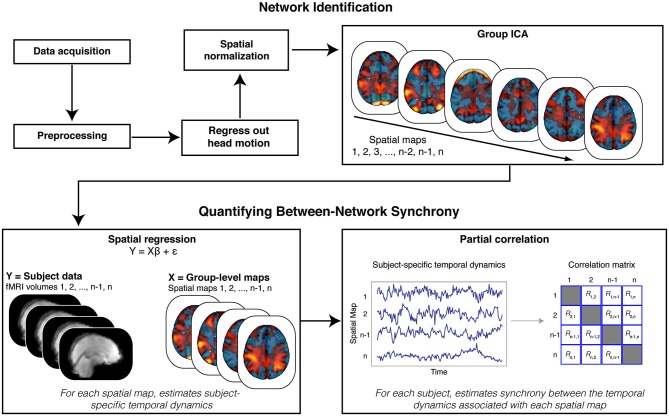
**Analysis Schematic**. Our analysis proceeded in multiple steps. Data were first preprocessed, and motion parameters and volumes identified as motion spikes were regressed from the data. Filtered data were then spatially transformed into standard (MNI) space. Next, we stacked data across participants and submitted the resulting matrix to a tensorial group independent component analysis (ICA). The ICA produced spatial maps that were then back projected onto the functional data (via spatial regression) to reveal participant-specific time courses for each spatial map. We estimated synchrony between the time courses associated with the spatial maps using partial correlation analyses. Crucially, partial correlation analysis allowed us to quantify, within each participant, the synchrony between each spatial map while controlling for the influence of other, potentially confounding (e.g., head motion), spatial maps.

We evaluated whether reduced connectivity between a sensory network related to perception of faces relative to scenes face-scene network (FSN) and a cognitive network containing the anterior cingulate cortex (executive control network (ECN; Smith et al., 2009)) predicted increased autistic traits. To test this hypothesis, we employed fMRI to record brain activation from 47 neurotypical males (i.e., individuals not exhibiting clinical symptoms of autism) viewing alternating blocks of faces and scenes (Figure 1); those individuals also completed the AQ as a behavioral measure of autism-spectrum traits. We used a model-free analytic technique (independent component analysis; ICA) (Beckmann and Smith, 2005) to identify key neural networks and to separate those networks from sources of unwanted variability (e.g., head motion). These networks were then submitted to a spatial regression to reveal how each network responds across time within each participant. Our key analysis focused on individual differences in the synchrony between ECN and FSN (Figure 2). We found that reduced synchrony between ECN and FSN correlated with greater severity in autistic traits (indicated by higher scores on the AQ). These findings suggest that changes in the interactions between large-scale neural networks may contribute to the pattern of altered function observed in individuals along the autism spectrum.

## Materials and Methods

### Participants

Forty-seven male young adults completed the study. Prescreening excluded individuals with prior neurological or psychiatric illness. We also excluded three participants prior to data analysis for excessive head motion (see below for motion quality control description), leaving a final sample of 44 for all analyses (mean age: 24.07 years; range: 18–32 years). All procedures and methods were conducted in accordance with guidelines approved by the Institutional Review Board at Duke University Medical Center. Written informed consent was obtained from all participants.

### Stimuli and Tasks

Prior to beginning the main task, all participants completed a variant of an unrelated response-time task using visual images (Libedinsky et al., 2011); results from this task will be reported elsewhere. The main task employed passive visual stimulation (Figure 1). Participants viewed eight blocks of face and scene images. Each block presented six images of the same category, but the attractiveness (determined by an independent group and unrelated to our current aims) could either be high or low. Images were presented for 3 s each and were separated by a 1 second fixation interval, yielding a total block length of 23 s for each block. Image blocks were separated by a 23 s fixation interval. To facilitate tensorial decomposition (Beckmann and Smith, 2005), all participants experienced the same stimuli in the same order: Face High Attractiveness, Scene Low Attractiveness, Face Low Attractiveness, Scene High Attractiveness, Scene Low Attractiveness, Face High Attractiveness, Face Low Attractiveness, Scene High Attractiveness. Once again, our key analyses focused on the image category rather than attractiveness.

After completing the scanner portion of the experiment, each participant completed two computer tasks. First, each participant completed a simple rating task where an attractiveness judgment was provided for each image previously seen in the response-time task and in passive viewing task. Each participant’s highest rated face and scene images were then used in a preference task. This task displayed 15 pairs of faces and scenes with identical high ratings, and the participant was asked to indicate which image he preferred. We used the relative number of face vs. scene choices as an idiosyncratic index of preferences; therefore, participants could range from −15 (complete scene preference) to 15 (complete face preference). All tasks were programed using the Psychophysics Toolbox (Version 3) (Brainard, 1997).

Each participant also completed the AQ (Baron-Cohen et al., 2001). As an additional control for personality variables related to social cognition, we also used the interpersonal reactivity index (Davis, 1983), which provided empathetic concern and perceptive-taking subscales.

### Image Acquisition

Images were acquired with a General Electric MR750 3.0 Tesla scanner equipped with an 8-channel parallel imaging system. To collect the neuroimaging data, we utilized a T_2_*-weighted spiral-in sensitivity encoding sequence (acceleration factor = 2), with slices parallel to the axial plane connecting the anterior and posterior commissures (repetition time (TR): 1580 ms; echo time (TE): 30 ms; matrix: 64 × 64; field of view (FOV): 243 mm; voxel size: 3.8 × 3.8 × 3.8 mm; 37 interleaved axial slices acquired in ascending order; flip angle: 70 degrees). The first eight volumes were discarded prior to preprocessing the functional data. To facilitate coregistration and normalization of the functional data, we also acquired whole-brain high-resolution anatomical scans (T_1_-weighted FSPGR sequence; TR: 7.58 ms; TE: 2.93 ms; matrix: 256 × 256; FOV: 256 mm; voxel size: 1 × 1 × 1 mm; 206 axial slices; flip angle: 12 degrees).

### FMRI Preprocessing

We used the FMRIB Software Library (FSL Version 4.1.8)^1^ package (Smith et al., 2004) for preprocessing. We first motion corrected our data by realigning the time series to the middle volume (Jenkinson et al., 2002). We then skull stripped the non-brain material using the brain extraction tool (Smith, 2002) and corrected for intravolume slice-timing differences using Fourier-space phase shifting to align to the middle slice. After spatially smoothing the images with a 5 mm full-width-half-maximum Gaussian kernel, we applied a high-pass temporal filter to remove signals with periods exceeding 100 s. Finally, each 4-dimensional dataset was grand-mean intensity normalized using a single multiplicative factor. Prior to group analyses, functional data were spatially normalized to the Montreal Neurological Template (MNI) avg152 T_1_-weighted template (3 mm isotropic resolution) using a 12-parameter affine transformation implemented in FLIRT (Jenkinson and Smith, 2001).

As part of our preprocessing, we also examined three partially correlated metrics summarizing participant-specific data quality: signal-to-fluctuation-noise ratio (SFNR; Friedman and Glover, 2006), mean volume-to-volume head motion, and number of motion spikes within the time series. To identify volumes as motion spikes, we first computed the root-mean-square error (RMSE) of each volume relative to the middle time point; the resulting values were then submitted to a boxplot threshold to identify outliers (i.e., RMSE amplitude exceeded the 75th percentile plus the value of 150% of the interquartile range of RMSE for all volumes in a run). We excluded subjects where any of these measures was extreme relative to other subjects (i.e., beyond the upper or lower 5th percentile in the distribution of values for that specific measure). This procedure created the following exclusion thresholds: SFNR < 34.68; proportion of outlier volumes > 0.121; mean volume-to-volume head motion > 0.204 mm. These thresholds identified three problematic subjects who were excluded from further analysis, leaving a final sample of 44 subjects for all analyses. To improve the data quality in the remaining subjects, we also regressed out variance tied to the motion parameters and also the motion spikes.

### General Linear Model

We used FEAT to estimate a general linear model with local autocorrelation. The model included four regressors corresponding to the blocks of image presentation (Face High Attractiveness, Face Low Attractiveness, Scene High Attractiveness, and Scene Low Attractiveness). We also included covariates associated with motion parameters and motion spikes. Our analysis focused on a bidirectional contrast of face blocks relative to scene blocks. We combined data across participants using a mixed-effects model (Beckmann et al., 2003). Brain activations are displayed using MRIcroGL,^2^ and anatomical labels for local maxima (all coordinates reported in MNI space) were obtained using the Harvard-Oxford Cortical and Subcortical atlases (Zilles and Amunts, 2010).

### Independent Component Analyses

To identify large-scale neural networks in the fMRI data, we performed a tensor-based independent components analysis (ICA) using Multivariate Exploratory Linear Decomposition into Independent Components (MELODIC) Version 3.10 within FS (Beckmann and Smith, 2004, 2005). Prior to estimating the ICAs, we demeaned each participant’s functional data and normalized the voxel-wise variance to prevent regions with high variability (e.g., cerebrospinal fluid) from biasing the ICA (Beckmann and Smith, 2004). The resulting dataset was then whitened and projected into a 25-dimensional subspace using probabilistic principal component analysis. Using a fixed-point iteration technique (Hyvärinen, 1999) to optimize for non-Gaussian spatial source distributions, the whitened observations were decomposed into sets of vectors that describe signal variation across the temporal, spatial, and subject domains (Beckmann and Smith, 2005). The estimated component maps were thresholded by dividing the maps by standard deviation of the residual noise and then fitting a Gaussian-Gamma mixture model to the histogram of normalized intensity values (Beckmann and Smith, 2004).

### Dual-Regression Analyses

To evaluate individual differences in connectivity with spatial maps identified by the ICA, we employed a dual-regression analytical approach (Filippini et al., 2009; Murty et al., 2014; Smith et al., 2014b; Utevsky et al., 2014). The dual-regression approach first requires a *spatial-regression*. In this step, spatial maps are regressed onto each participant’s functional data, resulting in a T (time points) × C (components) set of beta coefficients that characterize the within-subject temporal dynamics of each spatial network. In the second *temporal-regression* step, the resulting temporal dynamics from the first step that describe each network, in each subject, are regressed onto each subject’s functional data. The output of this analysis is a set of spatial maps that quantify each voxel’s connectivity with each network identified with the group ICA, individually for each subject. Importantly, this analysis estimates each voxel’s connectivity with each spatial network while controlling for the influence of other networks—some of which may reflect artifacts, such as head motion and physiological noise.

### Quantifying Synchrony Between Networks

We adopted a synchrony analysis based on previous research (Cole et al., 2010). We focused our core analyses on the synchrony between the ECN and the FSN. First, we used a spatial correlation to select the independent component map that best matched the network defined as the ECN in prior work (Smith et al., 2009),^3^ and the map that best matched the contrast for faces relative to scenes from our GLM model, respectively. Next, the entire set of independent component maps were regressed onto the functional data to reveal participant-specific time courses for each map. These time courses were then submitted to a partial correlation analysis to estimate the synchrony between each network, after accounting for correlations with all other networks (Figure 2). This technique allowed us to control for the influence of shared variance with the other independent components in the functional data, as well as other potential confounds (e.g., head motion). Taken together, this analytical approach is analogous to a between-sessions psychophysical interaction analysis, where functional connectivity (between networks) can be tested for dependency on an inter-subject variable (e.g., AQ scores) (Friston, 2011; O’Reilly et al., 2012).

## Results

### Identifying Key Sensory and Cognitive Networks

Our analyses focused on the interaction between social and ECN. To identify these networks, we employed two approaches. First, we used a general linear model to compare responses to faces and scenes (see Section Materials and Methods). This analysis revealed canonical areas implicated in processing visual stimuli containing faces (Kanwisher et al., 1997) and scenes (Epstein and Kanwisher, 1998). Specifically, a comparison of responses evoked by viewing faces relative to those evoked by viewing scenes revealed activation in the fusiform face area (Table 1). Likewise, a comparison of responses evoked by viewing scenes relative to those evoked by viewing faces revealed activation in the parahippocampal place area (Table 2).

**Table 1:** **Regions responding to faces relative to scenes**.

Probabilistic anatomical label	*x*	*y*	*z*	*Z*-stat	Number of voxels *(p)*
iLOC (47%), OFG (6%)	48	−76	−16	6.91	10467 (*p* < 0.001)
iLOC (57%), OFG (5%)	42	−80	−12	6.81	
iLOC (47%), OFG (6%)	42	−76	−10	6.76	
OFG (39%), iLOC (28%)	40	−70	−18	6.71	
iLOC (57%), OFG (14%)	−40	−82	−16	6.61	
OFG (19%)	−40	−72	−22	6.5	
Amygdala (71%)	16	−6	−14	6.14	7189 (*p* < 0.001)
Amygdala (23%)	12	−4	−16	5.98	
Amygdala (6%)	18	0	−10	5.96	
Amygdala (22%)	−18	−2	−12	5.69	
aTFC (59%), pTFC (25%)	32	−6	−42	5.62	
Amygdala (80%), Hippocampus (9%)	−18	−10	−14	5.45	
ParaCG (18%), SFG (18%), Frontal Pole (15%)	0	54	22	4.39	1896 (*p* < 0.001)
Frontal Pole (22%)	−18	58	36	4.3	
Frontal Pole (45%)	−32	56	26	4.29	
ParaCG (10%), SFG (10%)	−10	52	22	4.25	

*Listed in the table are regions exhibiting greater activation for faces relative to scenes, as determined by our general linear model. For clarity, we omitted labels whose likelihood is less than 5%. Abbreviations: iLOC (lateral occipital cortex, inferior division); OFG (occipital fusiform gyrus); SFG (superior frontal gyrus); aTFC (temporal fusiform cortex, anterior division); pTFC (temporal fusiform cortex, posterior division); ParaCG (paracingulate gyrus)*.

**Table 2:** **Regions responding to scenes relative to faces**.

Probabilistic anatomical label	*x*	*y*	*z*	*Z*-stat	Number of voxels *(p)*
TOFC (60%), pTFC (13%), LG (5%)	−26	−48	−14	7.11	18018 (*p* < 0.001)
TOFC (35%), LG (23%), pTFC (20%), pPHG (11%)	26	−40	−14	7.08	
TOFC (65%), LG (13%)	30	−44	−12	7.07	
TOFC (56%)	30	−48	−12	7.07	
TOFC (50%)	−28	−54	−12	7.04	
pPHG (38%), pTFC (28%), LG (10%), TOFC (8%)	22	−36	−18	6.98	
SFG (25%), MFG (11%)	−22	6	50	5.09	1887 (*p* < 0.001)
SFG (40%), MFG (17%)	−24	4	58	4.5	
PG (8%), SFG (6%)	−24	−6	44	4.17	
SFG (24%), MFG (15%)	−24	18	42	3.87	
Frontal pole (52%)	−34	52	2	3.55	
Frontal pole (87%)	−42	50	8	3.54	
SFG (23%)	20	8	52	4.52	583 (*p* < 0.05)
SFG (44%), MFG (7%)	22	16	54	4.2	
SFG (39%), MFG (6%)	22	14	50	4.17	
SFG (66%)	20	12	66	3.32	

*Listed in the table are regions exhibiting greater activation for scenes relative to faces, as determined by our general linear model. For clarity, we omitted labels whose likelihood is less than 5%. Abbreviations: iLOC (lateral occipital cortex, inferior division); PG (precentral gyrus); OFG (occipital fusiform gyrus); SFG (superior frontal gyrus); MFG (middle frontal gyrus); TOFC (temporal occipital fusiform cortex); pTFC (temporal fusiform cortex, posterior division), LG (lingual gyrus), pPHG (parahippocampal gyrus, posterior division)*.

**Figure 3:**
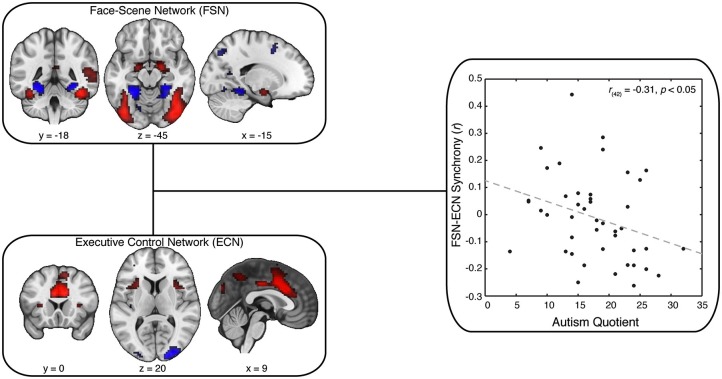
**Synchrony Between FSN and ECN Reflect Variation in AQ**. Our key analysis focused on synchrony between spatial maps postulated to involve face-scene processing (Face-Scene Network; FSN) and executive control (Executive Control Network; ECN). These maps were selected from our ICA spatial maps using spatial correlation: FSN was matched to the general-linear model output associated with a contrast of faces minus scenes; ECN was matched to an independent reference map derived from another study (Smith et al., 2009). Strikingly, we found that decreased synchrony between FSN and ECN predicted greater scores on the Autism Quotient (AQ). Crucially, this result was not explained by individual differences in head motion, data quality, or personality variables related to social cognition.

Next, we used independent components analysis (ICA) to identify distinct neural networks modulated by our task (Beckmann and Smith, 2004, 2005; Beckmann, 2012). This analysis produced a set of 25 spatial maps, with some maps resembling artifactual signals (e.g., head motion) and others reflecting cognitive and sensory networks. These independent component maps were then compared (using a spatial correlation analysis) against the contrast image associated with viewing faces relative to viewing scenes. We selected the independent component map with the highest spatial correlation with our contrast image (*r*_max_ = 0.71; other maps: *r*_mean_ = 0.03); hereafter, we refer to this map as our FSN (Figure 3, upper left). We applied a similar spatial correlation approach to identify the ECN identified in prior work (Smith et al., 2009). This canonical spatial map was similar to one independent component map in our data (*r*_max_ = 0.30; other maps: *r*_mean_ = 0.04), which will henceforth be referred to as our ECN (Figure 3, lower left). (We note that some groups label the ECN as a salience network; however, the precise name of the network does not impact our core conclusions because the two names describe the same neuroanatomical network shown in Figure 3).

### Reduced Synchrony between ECN and FSN Reflects Increased Autistic Traits

We next examined whether the ECN and FSN contribute to variation in autistic traits. We predicted autistic traits would be associated with the synchrony between the ECN and FSN. To test this prediction, we adopted a two-stage analytical approach (Figure 2; Cole et al., 2010). First, all spatial maps—those associated with signal and those associated with noise—are regressed onto each participant’s functional data to recover participant-specific temporal dynamics associated with each network. Second, these temporal dynamics are then submitted to a partial correlation analysis to estimate the participant-specific synchrony between each map while controlling for the influence of other, potentially confounding, maps. The resulting synchrony measures were then correlated with AQ scores. We found that reduced synchrony between ECN and FSN predicted increased autistic traits (*r*_(42)_ = −0.31, *p* < 0.05; Figure 3, right). To evaluate the uncertainty associated with this correlation, we bootstrapped the effect size (*N* = 10,000) and identified the 95% confidence interval, which was bounded by −0.05 and −0.55. Although this confidence interval indicates that the true effect is likely small, we emphasize that a relatively small effect is to be expected given a large sample and the use of measures with imperfect reliability (Vul et al., 2009; Yarkoni, 2009).

Thus, to increase our confidence in the validity of our effect and rule out potentially confounding variables we conducted a series of *post hoc* control analyses. We first examined the relationship between autistic traits and preferences for faces relative to scenes. Although most subjects exhibited a slight bias toward choosing faces over scenes (mean = 4.22; range = −15:15), these preferences were not correlated with AQ scores (*r*_(42)_ = 0.18, *p* = 0.24). Next, we evaluated whether the relationship between ECN-FSN synchrony and autistic traits was explained by individual differences in other, potentially confounding factors related to data quality and personality characteristics. To do this, we employed a hierarchical regression method that regressed ECN-FSN synchrony onto two blocks of factors. First, we included five regressors relating to individual differences in data quality (motion spikes and SFNR; Friedman and Glover, 2006), preferences between faces and scenes, and personality variables related to social cognition (perspective-taking abilities and empathic-concern) (Davis, 1983). This set of factors failed to explain significant variation in ECN-FSN synchrony (*r*^2^ = 0.02; *F*_(5,38)_ = 0.19, *p* = 0.96). Second, we added AQ scores to our regression model. We found that the addition of AQ scores significantly improved the fit our model (Δ *r*^2^ = 0.10; *F*_(1,37)_ = 4.30, *p* < 0.05). In addition, AQ scores were significantly associated with ECN-FSN synchrony (*t*_(37)_ = −2.07, *p* < 0.05) (Table 3).

**Table 3:** **Regression statistics**.

Regressor	Beta	VIF	Standard error	*t*-stat	*P*-value
Motion spikes	−1.0141	1.85	1.2740	−0.80	0.431
SFNR	−0.0003	1.74	0.0014	−0.20	0.839
Face-scene preference	0.0017	1.06	0.0034	0.51	0.616
Perspective-taking	0.0008	1.25	0.0060	0.14	0.891
Empathy	0.0038	1.21	0.0065	0.58	0.565
Autism-Spectrum	−0.0089	1.20	0.0043	−2.07	0.045
quotient

*The full regression model included six regressors and an intercept. Supporting our key finding, we found that synchrony between FSN and ECN was significantly associated with variation in AQ scores, even after controlling for potentially confounding variables. We also summarize the severity of multicollinearity for each regressor using variance inflation factor (VIF). As all VIF values are <5, we can be confident that multicollinearity did not bias our results (Kutner et al., 2004)*.

In a separate *post hoc* control analysis, we also examined the specificity of the relationship between AQ scores and ECN-FSN synchrony. Although our partial correlation analysis (Figure 2) estimates inter-network synchrony while controlling for the influence of potentially confounding variables (e.g., other networks), it remains possible that the relationship between AQ scores and network synchrony is driven by responses to the task. To rule out this explanation, we quantified synchrony between the FSN and the default-mode network (DMN), an alternative large-scale network implicated in a host of cognitive processes (Buckner et al., 2008; Mars et al., 2012). We found that FSN-DMN synchrony was not associated with variation in AQ scores (*r*_(42)_ = 0.01, *p* = 0.95). This observation therefore increases confidence in the specificity of our key finding.

For completeness, we also examined whether AQ scores significantly modulated voxelwise functional connectivity with either ECN or FSN. To test this relationship, we expanded our synchrony analysis into a dual-regression analysis by regressing the time course of each voxel onto the temporal dynamics for all networks (Filippini et al., 2009; Smith et al., 2014b; Utevsky et al., 2014) (see Section Methods). This analysis failed to reveal any voxels whose functional connectivity with FSN or ECN decreased (or increased) as a function of AQ scores. Although it is challenging to interpret the absence of an effect, these observations suggest that the computations related to AQ and tied to ECN and FSN are distributed across each network (Friston, 2011; Smith et al., 2014b).

## Discussion

Navigating social interactions is essential in everyday life, yet even basic social interactions can pose tremendous difficulty for individuals with ASD—and even for individuals who lack a formal diagnosis of autism but who share autism spectrum traits. Researchers investigating the neural bases of autism have consistently highlighted the importance of atypical connectivity (Supekar et al., 2013), particularly underconnectivity (i.e., reduced connectivity) across brain regions (Just et al., 2004, 2007). We predicted that increased autistic traits would be associated with reduced synchrony between two distinct neural networks implicated in cognitive control and face processing. To test this prediction, we used a model-free ICA in conjunction with a partial correlation analysis to characterize synchrony between large-scale neural networks. We found that reduced synchrony between the ECN and a FSN correlated with increased AQ scores within a subclinical population.

Our findings are consistent with previous work linking underconnectivity to autistic traits (Just et al., 2007; Di Martino et al., 2011; Müller et al., 2011). Reduced connectivity has reliably been observed with regions involved in face and visual information processing (Villalobos et al., 2005; Kleinhans et al., 2008). In addition, tasks requiring executive control have revealed that individuals with autism exhibit reduced connectivity with dorsolateral prefrontal cortex (Koshino et al., 2005). More recent observations have suggested that increased autistic traits are also associated with reduced frontostriatal connectivity (Sims et al., 2014). Although these diverse findings hint that neither autism nor autistic spectrum traits result from a single factor in isolation (Happé et al., 2006), recent work has attempted to understand how underconnectivity disrupts the integration of information (Just et al., 2004, 2007). Accordingly, reduced synchrony between the ECN and FSN networks may suggest difficulty in integrating information across sensory and cognitive processes. Nevertheless, we note that other interpretations are also possible. For example, the association between ECN-FSN synchrony and autistic traits might reflect an individual’s ability to discern the emotional states of the faces (Alaerts et al., 2014). Alternatively, reduced synchrony between ECN and FSN could represent a decrease in the reward valuation and processing of these facial images (Sims et al., 2014). While more work is needed to discern exactly how individual differences in ECN-FSN connectivity are influencing the behavioral and personality traits seen in autistic individuals, we believe our findings fit with other studies linking underconnectivity to autistic traits.

Unlike the majority of previous work examining functional underconnectivity and autistic traits, we focused on reduced connectivity between distinct, large-scale neural networks. Our approach allowed us to estimate how interactions between the ECN and FSN contribute to variation in autistic traits while controlling for potential confounds related to head motion. Controlling for head motion is crucial in functional connectivity studies (Power et al., 2012), particularly those examining between-subject differences (Satterthwaite et al., 2012), such as variation in autistic traits. Additionally, our study also expands on the findings of prior observations that have been limited to resting-state functional connectivity. Although one previous study has examined resting-state connectivity between networks in autistic individuals (von dem Hagen et al., 2013), our task utilized alternating blocks of social and nonsocial visual stimulation to drive changes in processing, which may be an important consideration given the social deficits observed in individual exhibiting a range of subclinical and clinical autistic traits. Yet, we note that directly linking task conditions (e.g., social and nonsocial) to reduced synchrony would require a resting-state control scan (Utevsky et al., 2014) and/or an alternative paradigm amenable to modeling task-dependent changes in connectivity using a beta-series correlation approach (Rissman et al., 2004). While these considerations could help illuminate how task conditions influence synchrony, we emphasize that our core conclusion is agnostic on the role of task conditions and network synchrony. Taken together, our results complement and extend prior work examining how reduced connectivity contributes to a range of autistic traits.

Nevertheless, we note that some limitations accompany our results. First, we did not examine task-dependent changes in connectivity in this study. Thus, our synchrony results—like those reported elsewhere (Cole et al., 2010)—could partially reflect changes in the activation of each network (Friston, 2011). Also, because our FSN arises because of differences in the brain responses to faces and scenes, we cannot be sure that our results are driven by neural processing specific to faces. Future work could build on our results by isolating specific features or qualities of facial stimuli—such as their emotional expression (Ashwin et al., 2007), attractiveness (Smith et al., 2014a), or perceived status (Utevsky and Platt, 2014)—and showing that variability in those features drives the connectivity changes we observed. In addition, although we controlled for head motion and other potential confounds, we note that other, unmeasured factors may contribute to our findings. For example, AQ scores have been linked to other personality variables, including extraversion, neuroticism, and conscientiousness (Wakabayashi et al., 2006). Other studies have also found associations between AQ and empathic abilities and systemizing tendencies (Wheelwright et al., 2006) as well as social responsiveness (Armstrong and Iarocci, 2013). Although we did not measure these variables in our study, we emphasize that our results were not explained by individual differences in empathetic concern or perceptive-taking subscales of the interpersonal reactivity index (Davis, 1983). Future studies could further mitigate these caveats with larger (and more diverse) samples and a larger array of personality measures.

In summary, our results suggest that the synchrony between large-scale neural networks can contribute to the severity of subclinical autistic traits, as measured by the AQ. Examining synchrony between large-scale neural networks may provide enhanced sensitivity for detecting subtle abnormalities associated with autistic traits (Tyszka et al., 2014). We speculate that the relationship between large-scale neural networks could have broad implications for social and cognitive neuroscience. For instance, network synchrony measures may facilitate comparative studies of social cognition by grounding questions on the dynamics of large-scale neural networks (Hecht et al., 2013). Additionally, our analytic approach may have implications for understanding other disorders as well. For example, it remains unclear how the synchrony between large-scale neural networks contributes to depression (Greicius et al., 2007), schizophrenia (Calhoun et al., 2009), and anorexia (Watson et al., 2010). In the future, clinicians could potentially use network synchrony measures as a biomarker for the severity of various personality disorders.

## Human Subjects Certification

All procedures and methods were conducted in accordance with guidelines approved by the Institutional Review Board at Duke University Medical Center. Written informed consent was obtained from all participants (text is reproduced in Section Methods).

## Author Contributions

DVS, JSY, CGC, and SAH designed research; DVS, JSY, CGC, and SAH performed research; DVS and JSY analyzed data under the supervision of SAH; DVS, JSY, CGC, and SAH wrote the paper.

## Conflict of Interest Statement

The authors declare that the research was conducted in the absence of any commercial or financial relationships that could be construed as a potential conflict of interest.

## Footnotes

^1^http://www.fmrib.ox.ac.uk/fsl/

^2^http://www.mccauslandcenter.sc.edu/mricrogl/

^3^See http://fsl.fmrib.ox.ac.uk/analysis/brainmap+rsns/
